# Leiomyosarcoma of the inferior vena cava: A case report of a rare tumor entity

**DOI:** 10.1016/j.ijscr.2020.04.094

**Published:** 2020-05-13

**Authors:** Kira Keller, Barbara Jacobi, Mahmoud Jabal, Gregor Alexander Stavrou

**Affiliations:** Department of General, Visceral, Thoracic, and Oncologic Surgery, Klinikum Saarbrücken, Germany

**Keywords:** Leiomyosarcoma, Sarcoma, Vena cava, Case report

## Abstract

**Introduction:**

Leiomyosarcomas are rare and heterogeneous group of tumors that account for less than 1% of adult malignancies. More than 50% of all vascular leiomyosarcomas occur in the lower part of vena cava. Since the first description of Perl and Virchow in 1871, only approximately 450 cases have been reported in literature.

**Presentation of Case:**

The patient presented due to abdominal pain and weight loss. Based on the imaging evaluations a retroduodenal tumor with compression of the inferior vena cava was observed. In the explorative laparotomy a leiomyosarcoma originating from the inferior vena cava was identified. Considering the extensive intramural and intraluminal tumor manifestation, the patient underwent a segmental resection of the vena cava. Reconstruction was achieved by implanting a polytetrafluoroethylene (PTFE) prosthesis. Postoperatively a stenosis developed due to a pericaval haematoma with consecutive compression of the prosthesis. An angiographic implantation of a stent was successfully performed. In the 24-month follow-up, the patient is free of symptoms and tumors.

**Discussion:**

Leiomyosarcomas of the vena cava are classified anatomically according to their relationship to the liver and renal vessels. The clinical symptoms depend on the affected segment. The therapy of choice is radical en bloc tumor resection. After resection, the options for reconstruction include placement of a synthetic graft, primary repair and patch repair.

**Conclusion:**

Due to a variety of topographic and tumor biological sarcoma manifestations, no standard has been established for the resection of this entity. The extent of resection should be planned individually.

## Highlights

• Leiomyosarcoma is a rare tumor accounting for less than 1% of adult malignancies.
• So far, only approximately 450 cases have been reported in literature.
• Surgical resection is the treatment of choice.
• Case report of a patient with leiomyosarcoma originating from the inferior vena cava.
• The patient is free of disease recurrence 24 months after surgery.

## 1 Introduction

Leiomyosarcomas are rare and heterogeneous group of tumors that account for less than 1% of adult malignancies [1]. More than 50% of all vascular leiomyosarcomas occur in the lower vena cava [2]. They grow from the tunica mucosa of the blood vessels.

Since the first description of Perl and Virchow in 1871 [3], only approximately 450 cases have been reported in literature [4]. The first surgical resection was performed by Mechior in 1928 [5].

Due to the slow growth, the tumors remain clinically inapparent for a long time and are usually diagnosed in a locally advanced tumor stage. This work has been reported in line with the SCARE criteria [6].

## 2 Methods

**Fig. 1:**
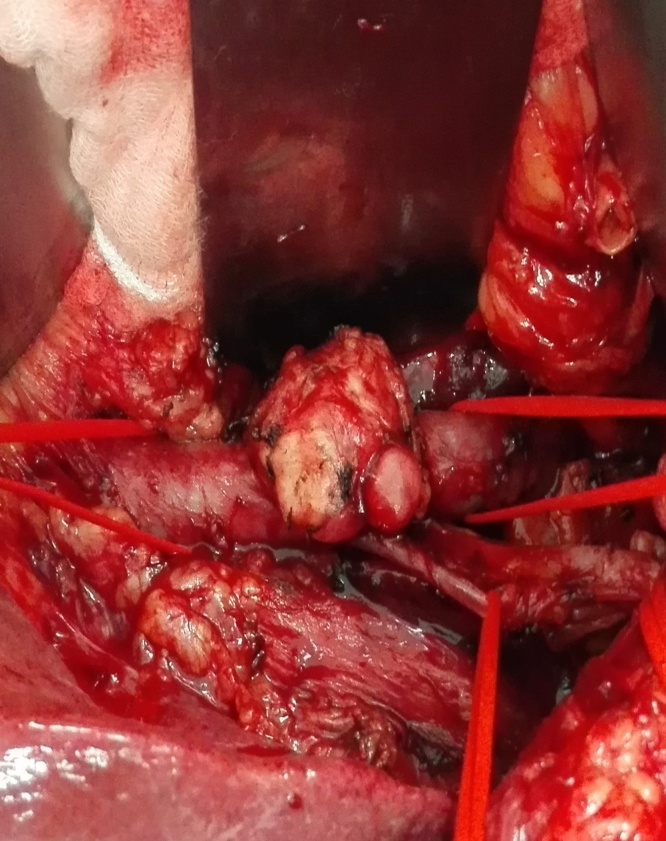
A discovered tumor located in inferior vena cava during intraoperative exploration.

**Fig. 2:**
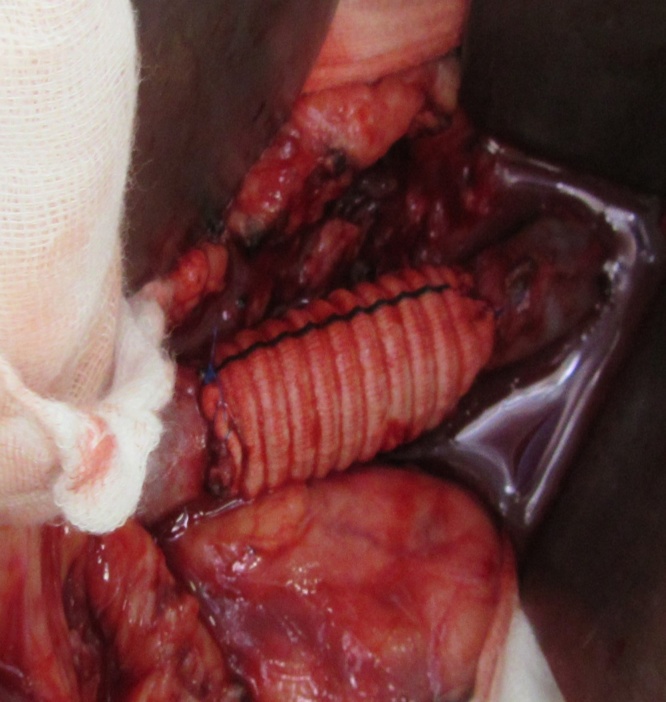
Segmental resection of the inferior vena cava and vascular reconstruction by implanting a polytetrafluoroethylene prosthesis.

The patient presented due to abdominal pain and weight loss. According to the preoperative computer tomography (CT) imaging evaluation, a retroduodenal tumor with compression of the inferior vena cava was observed. Clinical examinations did not show any sign of lower extremity edema or deep vein thrombosis. In the explorative laparotomy a tumor located in the infrarenal vena cava was identified (Fig. 1). Subsequently, a frozen section examination was performed, which demonstrated a neoplastic mesenchymal dysplasia. Considering the extensive intramural and intraluminal tumor manifestation, the patient underwent a segmental resection of the vena cava. Reconstruction was achieved by implanting a polytetrafluoroethylene (PTFE) prosthesis (Fig. 2).

## 3 Results

**Fig. 3:**
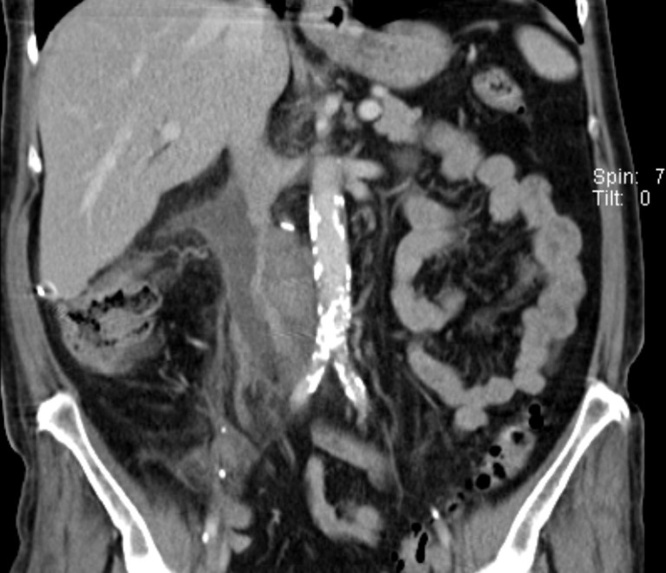
Stenosis developed due to a pericaval haematoma with consecutive compression of the prosthesis.

**Fig. 4:**
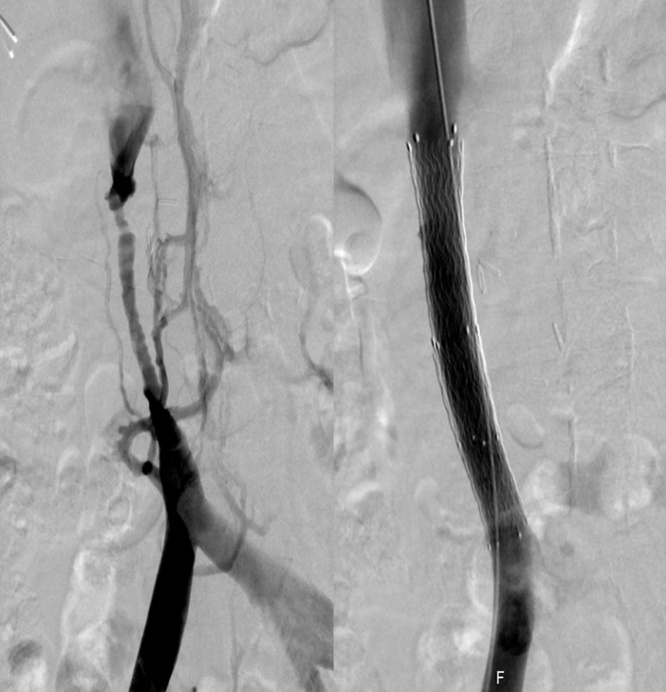
A successful angiographic implantation of a stent.

**Fig. 5:**
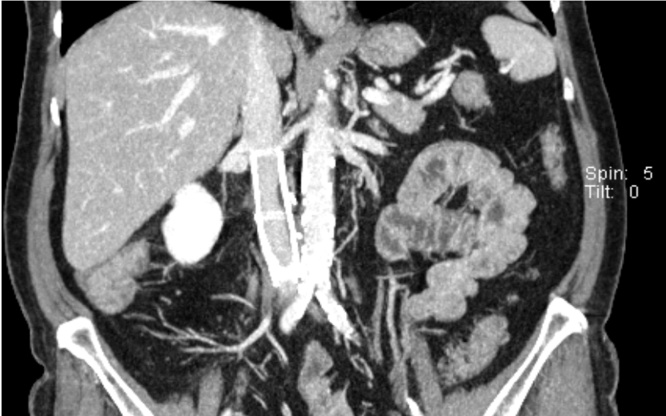
CT evaluation showing no sign of local/systemic disease recurrence 24 months after operation.

Postoperative histopathological evaluations identified a leiomyosarcoma originating from the inferior vena cava (grad 2 based on the FNCLCC classification) with a tumor stage of pT1b V1 R1 M0. Postoperatively the patient suffered from an abdominal pain. Subsequently, she underwent CT examination, which showed a stenosis developed due to a pericaval haematoma with consecutive compression of the prosthesis (Fig. 3). To resolve the stenosis, an angiographic implantation of a stent was successfully performed (Fig. 4). Due to the positive vascular resection margin, the patient did not receive any adjuvant therapy. However, we closely followed up the patient every 6 months with CT evaluations. After 24-month follow-up, the patient is free of symptoms and local/systemic disease recurrence (Fig. 5).

## 4 Discussion

Leiomyosarcomas of the vena cava are classified anatomically according to their relationship to the liver and renal vessels. The clinical symptoms depend on the affected segment. Segment I (caudal to the renal vessels) will cause lower extremity edema, deep vein thrombosis and abdominal pain; segment II (between renal and hepatic veins) causes nephrotic syndrome and abdominal pain; and segment III (suprahepatic to right atrium) causes weight loss, nausea, Budd-Chiari syndrome and cardiac arrhythmia [7,8]. Despite modern imaging techniques, the diagnosis can often only be made perioperativly.

Leiomyosarcomas originated from vena cava are the extremely rare diseases. Based on the available literature, the radical en bloc tumor resection seems to be the treatment of choice as the only therapeutic approach that can improve patient survival [9]. R0 resection can allow 5-year survival rates of nearly 50% [10]. Despite the promising results from the case series with very small number of patients underwent combination therapy with chemo/radiotherapy and surgery, the exact success rate of combination therapy is still unclear [11,12]. Therefore, this needs to be further evaluated in well-designed and large-scale prospective studies.

After resection, the options for reconstruction include placement of a synthetic graft, primary repair and patch repair. According to the literature the material of choice for a caval replacement is PTFE [[13], [14], [15]]. Studies also showed a graft patency of 67% after five years [16]. Graft occlusion was observed in 39% of cases [17].

## 5 Conclusion

Due to a variety of topographic and tumor biological sarcoma manifestations, no standard has been established for the resection of this entity. Taking these characteristics into account, the extent of resection should be planned individually.

## Funding

This study is not funded

## Ethical approval

This is a case report. Therefore, an ethical approval is not required.

## Consent

Written informed consent was obtained from the patient for publication of this case report and accompanying images. A copy of the written consent is available for review by the Editor-in-Chief of this journal on request.

## Author contribution

- The conception and design of the study: KK and GAS
- Acquisition of data: BJ and MJ
- Drafting the article: KK and MJ
- Revising it critically for important intellectual content: BJ and GAS
- final approval: All authors

## Registration of research studies

NA.

## Guarantor

Dr. Kira Keller

Winterberg 1, 66119 Saarbrücken, Germany, Phone: +49 (0)6 81 963 34168, Fax: +49 (0)6 681 963 2417, kikeller@klinikum-saarbruecken.de

## Informed consent

Written informed consent was obtained from the patient for publication of this case report and accompanying images. A copy of the written consent is available for review by the Editor-in-Chief of this journal on request.

## Provenance and peer review

Not commissioned, externally peer-reviewed.

## Declaration of Competing Interest

The authors declare no conflict of interest.
